# WSB1-Mediated PSMA Ubiquitination Promotes Enzalutamide-Induced Neuroendocrine-like Transition in Patient-Derived Prostate Cancer Spheroids

**DOI:** 10.3390/ijms27156899

**Published:** 2026-08-01

**Authors:** Dawa Jung, Ayse Tuba Kendi, David A. Woodrum, Daniel A. Adamo, Scott M. Thompson, Myung-Ho In, Gokce Belge Bilgin, Derek R. Johnson, Ian M. Horn, Eun-Joo Kim, Jin Ook Chung, Seon-Young Park, Geoffry L. Curran, Val J. Lowe, SeungBaek Lee

**Affiliations:** 1 Department of Radiology, Mayo Clinic, Rochester, MN 55905, USA; jung.dawa@mayo.edu (D.J.); kendi.ayse@mayo.edu (A.T.K.); woodrum.david@mayo.edu (D.A.W.); adamo.daniel@mayo.edu (D.A.A.); thompson.scott@mayo.edu (S.M.T.); in.myungho@mayo.edu (M.-H.I.); belgebilgin.gokce@mayo.edu (G.B.B.); johnson.derek1@mayo.edu (D.R.J.); horn.lan@mayo.edu (I.M.H.); curran.geoffry@mayo.edu (G.L.C.); 2 Department of Molecular Biology, Dankook University, Cheonan 31116, Republic of Korea; nbrejk@dankook.ac.kr; 3 Department of Internal Medicine, Chonnam National University Medical School, Gwangju 501757, Republic of Korea; imagine-jjo@hanmail.net (J.O.C.); drpsy@naver.com (S.-Y.P.); 4 Department of Molecular Pharmacology and Experimental Therapeutics, Mayo Clinic, Rochester, MN 55905, USA

**Keywords:** prostate cancer, patient-derived spheroids, enzalutamide resistance, treatment-emergent neuroendocrine prostate cancer, WSB1, prostate-specific membrane antigen, Aurora kinase A

## Abstract

Long-established prostate cancer cell lines provide limited insight into how contemporary early prostate cancer evolves into drug-resistant and neuroendocrine-like refractory disease. Patient-derived three-dimensional (3D) *ex vivo* models may better preserve this transition. Here, we found that 8 of 10 magnetic resonance imaging-guided biopsy specimens from patients with early-stage prostate cancer generated sustained 3D tumor spheroid cultures. After 12 weeks of enzalutamide selection, only one patient-derived culture acquired a resistant phenotype with treatment-emergent neuroendocrine prostate cancer (t-NEPC)-like features, including increased chromogranin A (CgA) and synaptophysin (SYP); reduced androgen receptor (AR), prostate-specific antigen (PSA), and prostate-specific membrane antigen (PSMA); and conversion from compact spheroids into irregular resistant aggregates. During this transition, WD repeat and SOCS box-containing protein 1 (WSB1) increased, whereas PSMA progressively decreased. *WSB1* silencing restored PSMA, AR, and PSA expression and reduced neuroendocrine-associated features. A similar WSB1 dependency was observed in enzalutamide-resistant LNCaP cells, the castration-resistant prostate cancer model 22Rv1, and the neuroendocrine/small-cell prostate cancer model NCI-H660. Mechanistically, WSB1 functioned as a SOCS box-dependent E3 ubiquitin ligase adaptor that promoted PSMA ubiquitination and degradation. SOCS box deletion or T380A mutation impaired this process, while Aurora kinase A (AURKA) inhibition reduced WSB1-dependent PSMA ubiquitination. *WSB1* depletion, AURKA inhibition with alisertib, and combined AURKA inhibition with *EZH2* suppression reduced resistant aggregate growth and increased apoptosis-associated markers in patient-derived enzalutamide-resistant neuroendocrine-like spheroids and related models. These findings nominate the AURKA–WSB1–PSMA axis as a therapeutic vulnerability in refractory prostate cancer.

## 1. Introduction

Prostate cancer represents a substantial global health burden. In the United States, an estimated 299,010 new cases and 35,250 deaths were projected for 2024 [1], while approximately 1.5 million new cases and 397,000 deaths were estimated worldwide in 2022, making prostate cancer the second most frequently diagnosed cancer and the fifth leading cause of cancer-related death among men globally [2]. Most prostate adenocarcinomas initially depend on androgen receptor (AR) signaling, providing the biological basis for androgen deprivation therapy and next-generation AR pathway inhibitors such as enzalutamide and abiraterone [3,4,5,6]. However, sustained AR pathway inhibition imposes strong therapeutic selection pressure, and many tumors ultimately progress to castration-resistant prostate cancer (CRPC) through AR-dependent or AR-independent resistance mechanisms, including lineage-infidelity states that reduce dependence on canonical AR signaling [7,8,9]. Among AR-independent resistance states, treatment-emergent neuroendocrine prostate cancer (t-NEPC) represents a particularly aggressive form of lineage plasticity. t-NEPC commonly arises from conventional prostate adenocarcinoma under prolonged therapeutic pressure and is characterized by attenuated AR signaling, loss of luminal prostate identity, induction of neuroendocrine markers, rapid disease progression, and limited response to standard AR-directed therapies [10,11,12,13,14,15]. Genomic and functional studies have implicated tumor suppressor loss, MYCN activation, enhancer reprogramming, stemness programs, epigenetic remodeling, inflammatory JAK/STAT–FGFR signaling, FOXA2–KIT pathway activation, and Aurora kinase A (AURKA) signaling in this transition [11,12,13,14,15,16,17,18,19]. Nevertheless, the post-translational mechanisms that connect chronic AR pathway inhibition to early neuroendocrine-like remodeling remain insufficiently defined. This gap is clinically important because lineage plasticity can also eliminate therapeutic targets. Prostate-specific membrane antigen (PSMA), encoded by folate hydrolase 1 (FOLH1), is a clinically important surface protein used for prostate cancer molecular imaging and radioligand therapy [20,21,22,23,24,25,26,27]. PSMA-targeted positron emission tomography (PET) improves detection and staging of clinically significant or recurrent disease [23,24], whereas lutetium-177–PSMA-617 has improved outcomes in PSMA-positive metastatic CRPC in both previously taxane-treated and taxane-naïve treatment settings [25,26]. Conversely, PSMA-low or PSMA-heterogeneous tumors are less suitable for PSMA-directed theranostic strategies [27]. Neuroendocrine differentiation has been associated with PSMA suppression, suggesting that tumors progressing toward t-NEPC may lose a clinically actionable vulnerability [28].

Patient-derived three-dimensional (3D) tumor models offer a strategy to study treatment adaptation in a more clinically relevant setting than conventional two-dimensional (2D) culture systems [29,30,31,32,33]. This need is particularly acute in prostate cancer, where mechanisms of drug resistance and neuroendocrine transition have been studied largely in long-established cell lines, advanced disease specimens, or metastatic models rather than living tissue from contemporary early-stage tumors. Consequently, the early events by which treatment-naïve prostate adenocarcinoma adapts to AR pathway inhibition and progresses toward CRPC or t-NEPC-like states remain difficult to model directly. Viable 3D culture from small prostate biopsy tissues remains challenging because tissue volume, tumor-cell content, ischemic stress, and processing-related injury can limit culture success [29,30,31,32,33]. Magnetic resonance imaging (MRI)-guided biopsy improves sampling of clinically significant prostate cancer lesions and provides an opportunity to establish living tumor cultures directly from clinically defined early disease [34,35]. Accordingly, MRI-guided biopsy-derived spheroids provide a clinically anchored platform to interrogate early drug adaptation, resistant outgrowth, and lineage remodeling before overt CRPC or t-NEPC is clinically established.

WD repeat and SOCS box-containing protein 1 (WSB1) is a WD-repeat and suppressor of cytokine signaling (SOCS) box-containing adaptor protein that can participate in Cullin-RING E3 ubiquitin ligase complexes [36,37,38,39,40]. Through this E3 ligase adaptor function, WSB1 can connect substrate recognition to proteasomal degradation, thereby altering cellular programs that restrain malignant adaptation. WSB1 has been implicated in multiple cancer contexts. Prior work has linked WSB1 expression or activity to tumor development in pancreatic cancer, hepatocellular carcinoma, and salivary gland tumors, and to metastatic progression, hypoxia-associated programs, or adverse metastasis-free outcomes in melanoma, prostate cancer, urinary bladder cancer, breast cancer, colon cancer, lung adenocarcinoma, and non-small cell lung cancer specimens [41,42]. We previously showed that WSB1 promotes metastatic progression by targeting the von Hippel-Lindau tumor suppressor protein (pVHL) for proteasomal degradation, enhancing hypoxia-inducible factor (HIF)-associated tumor adaptation and invasion; WSB1 expression was also elevated in metastatic prostate cancer tissues compared with normal or primary prostate cancer tissues [41]. We also demonstrated that WSB1 promotes early tumorigenic progression by targeting ataxia telangiectasia mutated (ATM), a central DNA damage response kinase, for ubiquitin-mediated degradation, thereby enabling bypass of oncogene-induced senescence [42]. Together, these findings position WSB1 as a cancer-associated proteolytic regulator of malignant adaptation, survival, and progression. However, whether WSB1 controls prostate cancer lineage plasticity, PSMA stability, or therapy-induced neuroendocrine-like progression has remained unknown.

We hypothesized that chronic enzalutamide pressure activates a WSB1-dependent degradation program that suppresses PSMA and facilitates neuroendocrine-like transition. Given the established association of AURKA with aggressive variant prostate cancer and t-NEPC biology, we further examined whether AURKA-sensitive signaling regulates WSB1-dependent PSMA turnover [16,17,43,44,45,46]. Here, we establish patient-derived 3D prostate tumor spheroids from MRI-guided early prostate cancer biopsy tissues and identify an AURKA-sensitive WSB1–PSMA degradation axis associated with enzalutamide resistance and NEPC-like progression.

## 2. Results

### 2.1. MRI-Guided Biopsy Tissues from Early Prostate Cancer Generate Adenocarcinoma-like 3D Spheroids

**Figure 1:**
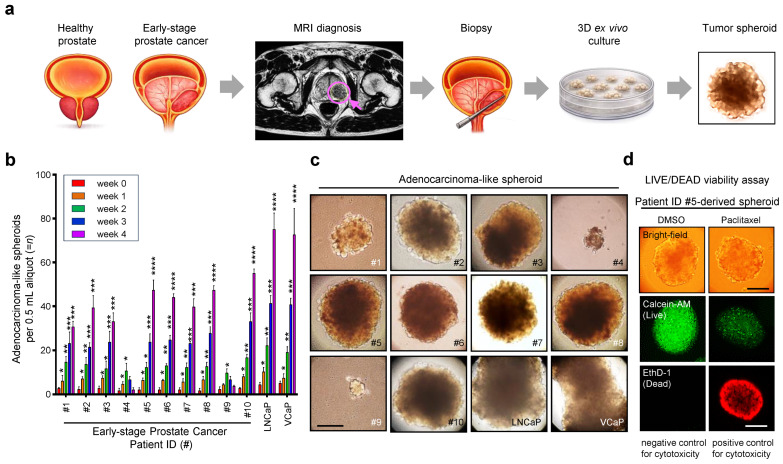
**Establishment of patient-derived prostate tumor spheroids from MRI-guided early prostate cancer biopsy tissues.** (**a**) Workflow for generating patient-derived prostate tumor spheroids from MRI-guided biopsy tissues. Fresh biopsy specimens were processed immediately for three-dimensional (3D) *ex vivo* culture. (**b**) Quantification of adenocarcinoma-like spheroids generated from patient-derived biopsy cultures over 4 weeks. Cultures were maintained in 6-well 3D culture plates using growth factor-enriched spheroid medium with partial conditioned-medium retention. LNCaP and VCaP spheroids were included as prostate adenocarcinoma cell line-derived controls. (**c**) Representative bright-field images of patient-derived prostate tumor spheroids and prostate cancer cell line-derived spheroid controls. Counted spheroids were defined as compact, rounded multicellular aggregates; single cells, debris, necrotic fragments, and loose non-compact clusters were excluded. (**d**) Representative bright-field and fluorescence images from the LIVE/DEAD viability assay of Patient ID #5-derived spheroids following vehicle (DMSO) or paclitaxel (Taxol; 10 nM for 48 h) treatment. Calcein-AM fluorescence identifies viable cells (green), whereas ethidium homodimer-1 (EthD-1) identifies membrane-compromised dead cells (red). Vehicle-treated spheroids exhibited predominant calcein-AM fluorescence with minimal EthD-1 signal. Taxol-treated spheroids showed reduced calcein-AM fluorescence and prominent EthD-1 staining and were included as a cytotoxic control. Quantitative data in panel b are shown as mean ± SEM from n = 3 independent experiments. Statistical significance in panel b was determined by two-way analysis of variance followed by Dunnett’s multiple-comparisons test relative to week 0 within each culture. * *p* < 0.05, ** *p* < 0.01, *** *p* < 0.001, and **** *p* < 0.0001. Scale bars, 50 µm.

Patient-derived organoid and spheroid platforms preserve tumor architecture, interpatient heterogeneity, drug response, and lineage states more effectively than conventional monolayer culture [29,30,31,32,33]. Because early prostate cancer biopsy tissues provide limited but clinically defined tumor material, we first asked whether specimens obtained through routine MRI-guided biopsy could be propagated as 3D tumor spheroids suitable for longitudinal drug-response studies [34,35]. Fourteen patients undergoing clinically indicated in-bore MRI-guided prostate biopsy for suspected or diagnosed early-stage prostate cancer were enrolled for research tissue collection. Biopsy procedures were performed using a combined targeted/systematic approach, and research cores were obtained from imaging-defined areas of clinical suspicion after completion of clinically required diagnostic sampling when feasible. Research biopsy material from 10 patients was ultimately available for ex vivo culture; specimens from four enrolled patients were not analyzed because clinical evaluation showed normal findings in some cases, biopsy scheduling was postponed in some cases, or an additional research core could not be provided because of limited biopsy core availability. Fresh research biopsy tissues were processed immediately after collection and placed into 3D ex vivo culture conditions (Figure 1a). Among the 10 evaluable MRI-guided biopsy specimens, 8 generated sustained 3D tumor spheroid cultures, corresponding to an 80% establishment rate under the tested workflow. During 4 weeks of culture, biopsy-derived tissues formed compact, rounded, multicellular structures with patient-to-patient variability in spheroid-forming capacity (Figure 1b). LNCaP and VCaP cells were included as prostate adenocarcinoma cell line controls and formed robust spheroids under the same workflow [47,48]. Representative bright-field images showed compact, multilayered tumor-like morphology in biopsy-derived cultures and cell line controls (Figure 1c). To directly assess the viability of the patient-derived structures classified as spheroids, representative Patient ID #5-derived spheroids were subjected to LIVE/DEAD fluorescence staining. Vehicle-treated spheroids exhibited intense calcein-AM fluorescence with minimal EthD-1 signal, indicating that they were composed predominantly of viable cells. In contrast, exposure to paclitaxel (Taxol; 10 nM for 48 h) reduced calcein-AM fluorescence and produced prominent EthD-1 staining, demonstrating the ability of the assay to detect treatment-induced cytotoxicity (Figure 1d). Together with sustained spheroid expansion over the 4-week culture period, these findings confirm that the representative Patient ID #5-derived spheroids examined were composed predominantly of viable cells and support the integrity of the biopsy-derived 3D culture platform. These data establish that clinically acquired MRI-guided prostate biopsy research cores can support biopsy-derived 3D prostate tumor spheroid generation for modeling early prostate cancer adaptation under therapeutic pressure.

### 2.2. Long-Term Enzalutamide Selection Identifies a Patient-Specific Resistant Spheroid Population with Neuroendocrine-like Features

**Figure 2:**
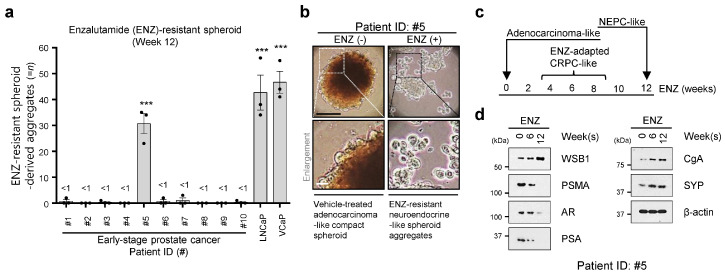
**Long-term enzalutamide selection identifies a patient-specific resistant spheroid-derived population with neuroendocrine-like features.** (**a**) Quantification of enzalutamide (ENZ)-resistant spheroid-derived aggregates after 12 weeks of ENZ treatment. Patient-derived prostate tumor spheroids were cultured with ENZ (10 µM), and resistant aggregates were counted at week 12. LNCaP and VCaP spheroids were included as prostate adenocarcinoma cell line-derived controls. (**b**) Representative bright-field images of Patient ID #5 cultures under vehicle control or ENZ-treated conditions. Upper panels show whole spheroid or aggregate morphology; lower panels show enlarged regions. (**c**) Schematic of the ENZ treatment time course. Patient ID #5 cultures were collected at weeks 0, 6, and 12 for molecular analysis. (**d**) Immunoblot analysis of WSB1, prostate-specific membrane antigen (PSMA), androgen receptor (AR), prostate-specific antigen (PSA), chromogranin A (CgA), synaptophysin (SYP), and β-actin in Patient ID #5 cultures during ENZ selection. β-actin was used as a loading control. Data in panel a are shown as mean ± SEM from n = 3 independent experiments. Statistical significance was determined by one-way analysis of variance followed by Tukey’s multiple-comparisons test. *** *p* < 0.001. Scale bars, 50 µm.

Enzalutamide is a potent AR pathway inhibitor, but durable response is limited by adaptive resistance and progression to CRPC [3,4,7]. Sustained AR pathway suppression can also drive lineage plasticity, allowing adenocarcinoma cells to acquire neuroendocrine-like traits associated with t-NEPC [10,11,12,13,14,15,16,17,18,19]. We therefore tested whether patient-derived early prostate cancer spheroids could evolve under chronic enzalutamide pressure. Biopsy-derived spheroids were cultured under continuous enzalutamide selection for 12 weeks. Most patient-derived cultures were drug-sensitive and failed to maintain spheroid growth. In contrast, Patient ID #5 reproducibly generated a distinct population of enzalutamide-resistant spheroid-derived aggregates at week 12 (Figure 2a). Vehicle-treated Patient ID #5 cultures maintained compact adenocarcinoma-like architecture, whereas enzalutamide-selected cultures lost spherical organization and formed irregular resistant aggregates (Figure 2b). To define the temporal molecular changes associated with this transition, cultures from Patient ID #5 were analyzed at weeks 0, 6, and 12 (Figure 2c). Week 0 spheroids retained PSMA, AR, and prostate-specific antigen (PSA). By week 6, WSB1 expression increased, while PSMA, AR, and PSA began to decline. By week 12, WSB1 was strongly induced, PSMA/AR/PSA were markedly reduced, and neuroendocrine-associated markers chromogranin A (CgA) and synaptophysin (SYP) increased (Figure 2d). These findings indicate a progressive enzalutamide-induced shift from an adenocarcinoma-like state toward an enzalutamide-resistant, t-NEPC-like phenotype.

### 2.3. WSB1 Depletion During Enzalutamide Adaptation Suppresses Resistant Aggregate Formation and Restores PSMA Expression

The time-course analysis of Patient ID #5 spheroids revealed progressive WSB1 induction during enzalutamide selection, coinciding with PSMA loss and acquisition of neuroendocrine-like features (Figure 2d). This temporal pattern suggested that WSB1 might be linked to the resistant PSMA-low state rather than serving only as a marker of drug adaptation. WSB1 is a SOCS box-containing E3 ubiquitin ligase adaptor capable of coupling substrate recognition to proteasomal degradation [36,37,38,39,40]. In previous studies, WSB1 promoted metastatic adaptation by targeting the von Hippel-Lindau tumor suppressor protein (pVHL) for degradation, thereby enhancing hypoxia-inducible factor (HIF)-associated tumor adaptation and invasion; WSB1 expression was also elevated in metastatic prostate cancer tissues compared with normal or primary prostate cancer tissues [41]. WSB1 was also shown to promote early tumorigenic progression by targeting ataxia telangiectasia mutated (ATM), a central DNA damage response kinase, for ubiquitin-mediated degradation, enabling premalignant cells to bypass oncogene-induced senescence [42]. These observations provided a rationale to test whether WSB1 contributes functionally to enzalutamide-resistant, PSMA-low prostate cancer spheroid growth.

**Figure 3:**
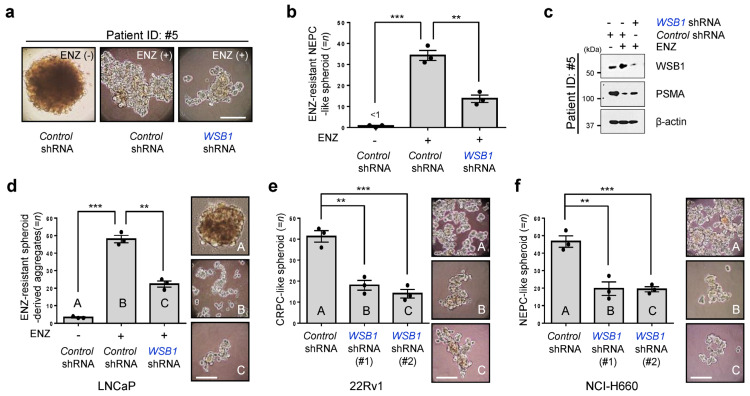
***WSB1* silencing reduces resistant spheroid and aggregate growth in patient-derived and prostate cancer reference models.** (**a**) Representative bright-field images of Patient ID #5-derived cultures transduced with non-targeting control or *WSB1*-targeting shRNA at week 6 of vehicle or continuous enzalutamide (ENZ) exposure and analyzed at week 12, six weeks after lentiviral transduction. (**b**) Quantification of ENZ-resistant spheroid-derived aggregates in Patient ID #5 cultures after control or *WSB1* knockdown. (**c**) Immunoblot analysis of WSB1 and PSMA in Patient ID #5 cultures after control or *WSB1* knockdown with or without ENZ treatment. β-actin was used as a loading control. (**d**) Representative images and quantification of LNCaP-derived ENZ-resistant aggregates after control or *WSB1* knockdown. (**e**) Representative images and quantification of 22Rv1-derived castration-resistant prostate cancer-like spheroids after control knockdown or two independent *WSB1*-targeting constructs. (**f**) Representative images and quantification of NCI-H660-derived neuroendocrine prostate cancer-like aggregates after control knockdown or two independent *WSB1*-targeting constructs. Control shRNA indicates non-targeting short hairpin RNA; *WSB1* shRNA indicates WSB1-targeting short hairpin RNA. Quantitative data are shown as mean ± SEM from n = 3 independent experiments. Statistical significance in panels (**b**,**d**–**f**) was determined by one-way analysis of variance followed by Tukey’s multiple-comparisons test. ** *p* < 0.01, and *** *p* < 0.001. Scale bars, 50 µm.

To test whether the emerging WSB1 induction contributed functionally to resistant progression, Patient ID #5-derived cultures were transduced at week 6 of continuous enzalutamide exposure, when WSB1 induction first became evident in the time-course analysis (Figure 2d), with lentiviruses encoding either non-targeting control or *WSB1*-targeting shRNA. The cultures were maintained under continuous enzalutamide pressure and analyzed at week 12, six weeks after lentiviral transduction. Under continued enzalutamide treatment, control shRNA-expressing cultures formed irregular resistant aggregates, whereas *WSB1* depletion markedly reduced aggregate formation (Figure 3a,b). Immunoblot analysis confirmed WSB1 loss and showed restoration of PSMA protein expression in enzalutamide-treated cultures (Figure 3c). These results suggest that WSB1 contributes, at least in part, to the progression of the early enzalutamide-adapted state toward a resistant PSMA-low phenotype in Patient ID #5-derived spheroids. We then examined whether this WSB1-associated dependency extended beyond the patient-derived resistant culture. *WSB1* silencing reduced aggregate formation in enzalutamide-resistant LNCaP cultures (Figure 3d). In 22Rv1 cells, an AR-positive CRPC-like model, two independent *WSB1*-targeting constructs decreased spheroid formation (Figure 3e) [49]. In NCI-H660 cells, an established neuroendocrine/small-cell prostate carcinoma model, WSB1 depletion also reduced neuroendocrine-like aggregate growth (Figure 3f) [50]. Collectively, these data suggest that WSB1 may support spheroid or aggregate growth across enzalutamide-resistant, CRPC-like, and neuroendocrine-like prostate cancer contexts, supporting WSB1 as a candidate contributor to PSMA-low-resistant progression.

### 2.4. WSB1 Promotes SOCS Box-Dependent PSMA Ubiquitination in LNCaP Cells

**Figure 4:**
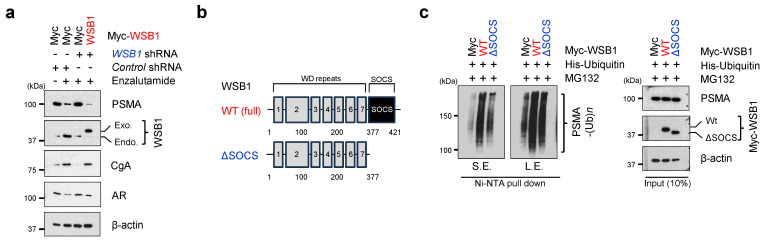
**WSB1 promotes PSMA ubiquitination in LNCaP cells through its SOCS box domain.** (**a**) Immunoblot analysis of PSMA, WSB1, CgA, AR, and β-actin in ENZ-treated LNCaP cells after control shRNA, *WSB1* shRNA, empty Myc vector, or Myc-WSB1 reconstitution, as indicated. Exogenous Myc-WSB1 and endogenous WSB1 bands are indicated. β-actin was used as a loading control. (**b**) Domain organization of wild-type WSB1 and the SOCS box deletion mutant. WD-repeat domains and the C-terminal suppressor of cytokine signaling (SOCS) box are indicated. (**c**) Denaturing His-ubiquitin pull-down assay for PSMA ubiquitination in LNCaP cells. Cells were transfected with His-ubiquitin together with an empty Myc vector, Myc-tagged WSB1 wild-type, or Myc-tagged WSB1 ΔSOCS constructs. MG132 was added before harvest. Ubiquitinated proteins were isolated using nickel-nitrilotriacetic acid (Ni-NTA) pull-down and immunoblotted for PSMA. Input lysates were analyzed for PSMA, Myc-tagged WSB1, and β-actin. S.E., short exposure; L.E., long exposure.

The restoration of PSMA after WSB1 silencing in Patient ID #5-derived resistant spheroids raised the possibility that WSB1 actively enforces the PSMA-low resistant state rather than passively accompanying lineage remodeling. To test the specificity and reversibility of this phenotype in a genetically tractable prostate adenocarcinoma model, we performed loss-of-function and rescue experiments in LNCaP cells, an AR-positive prostate cancer model widely used to study AR-regulated prostate lineage biology [47]. In enzalutamide-treated LNCaP cells, WSB1 suppression increased PSMA and AR expression while reducing CgA. Conversely, ectopic reconstitution with Myc-tagged WSB1 restored a PSMA-low, AR-low, CgA-high molecular profile (Figure 4a). These data support an on-target WSB1-dependent effect and indicate that WSB1 is sufficient to reimpose molecular features associated with enzalutamide-resistant neuroendocrine-like remodeling.

We next asked whether WSB1-mediated PSMA loss reflected ubiquitin-dependent protein turnover. WSB1 contains substrate-interacting WD-repeat domains and a C-terminal SOCS box that links substrate recognition to Elongin B/C-Cullin ubiquitin ligase complexes (Figure 4b) [36,37,38,39,40]. This architecture is consistent with prior WSB1 studies showing that WSB1 acts as a substrate-targeting E3 ligase adaptor for proteasomal degradation of pVHL and ATM [41,42]. We therefore tested whether PSMA is subject to WSB1-dependent ubiquitination during enzalutamide-associated lineage remodeling. Denaturing His-ubiquitin pull-down assays showed that wild-type WSB1 markedly increased high-molecular-weight ubiquitinated PSMA species in LNCaP cells (Figure 4c). In contrast, a SOCS box deletion mutant lacking the C-terminal Cullin-recruiting module strongly attenuated WSB1-driven PSMA ubiquitination (Figure 4b,c). These findings support a SOCS box-dependent E3 ligase adaptor mechanism through which WSB1 promotes PSMA ubiquitination, providing biochemical evidence that WSB1-associated proteolytic regulation may contribute to PSMA loss during enzalutamide-resistant neuroendocrine-like progression.

### 2.5. AURKA-Sensitive Signaling and the Conserved WSB1 T380 Region Modulate PSMA Ubiquitination

**Figure 5:**
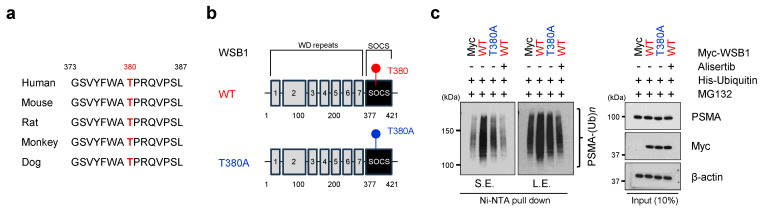
**AURKA inhibition and the conserved WSB1 T380 region modulate WSB1-dependent PSMA ubiquitination.** (**a**) Amino acid sequence alignment of the WSB1 region surrounding threonine 380 (T380) across human, mouse, rat, monkey, and dog WSB1 sequences. The conserved T380 residue is highlighted. (**b**) Domain organization of wild-type WSB1 and the phosphorylation-deficient T380A mutant. WD-repeat domains, the SOCS box, and the T380/T380A site are indicated. (**c**) Denaturing His-ubiquitin pull-down assay for PSMA ubiquitination in LNCaP cells expressing Myc-tagged WSB1 wild-type or T380A mutant constructs. Cells were transfected with His-ubiquitin together with an empty Myc vector, Myc-tagged WSB1 wild-type, or Myc-tagged WSB1 T380A constructs, with alisertib and MG132 added as indicated before harvest. Ubiquitinated proteins were isolated by Ni-NTA pull-down and immunoblotted for PSMA. Input lysates were analyzed for PSMA, Myc-tagged WSB1, and β-actin. S.E., short exposure; L.E., long exposure.

Having established that WSB1-mediated PSMA ubiquitination requires the SOCS box, we next examined whether this activity is regulated by phosphorylation-sensitive mechanisms. Phosphorylation–ubiquitination crosstalk allows kinase pathways to tune substrate recognition, E3 ligase adaptor activity, protein-complex assembly, and proteasomal degradation [51,52,53]. In prior WSB1 studies, phosphorylation of threonine 380 (T380) was linked to WSB1 activation, and mutation of this residue impaired WSB1-dependent ATM degradation during oncogene-induced senescence bypass [42]. Because AURKA has been associated with aggressive variant prostate cancer, MYCN-driven lineage plasticity, and t-NEPC biology, we tested whether the conserved WSB1 T380 region and AURKA-sensitive signaling regulate WSB1-mediated PSMA ubiquitination [16,17,43,44,45,46]. Cross-species alignment placed T380 within a conserved C-terminal regulatory region near the SOCS box, supporting a conservation-guided mutagenesis strategy (Figure 5a,b). In LNCaP cells, wild-type WSB1 robustly induced high-molecular-weight ubiquitinated PSMA species (Figure 5c). By contrast, the phosphorylation-deficient WSB1 T380A mutant showed a reduced capacity to promote PSMA ubiquitination, indicating that this conserved residue contributes to full WSB1 E3 ligase activity toward PSMA. Alisertib treatment also decreased WSB1-associated PSMA ubiquitination in cells expressing wild-type WSB1 (Figure 5c). These data identify the WSB1 T380 region as a regulatory element for PSMA ubiquitination and support a model in which AURKA-sensitive signaling modulates WSB1-dependent PSMA turnover during enzalutamide-resistant neuroendocrine-like progression.

### 2.6. AURKA Inhibition, WSB1 Suppression, and EZH2 Depletion Restrict Enzalutamide-Resistant NEPC-like Spheroid Growth

The preceding experiments linked enzalutamide-resistant neuroendocrine-like remodeling to WSB1 induction, PSMA loss, and SOCS box/T380-associated PSMA ubiquitination. We therefore asked whether this refractory spheroid state could be therapeutically challenged by perturbing WSB1-associated proteolytic regulation, AURKA-sensitive kinase signaling, or cooperating chromatin maintenance programs. This strategy was guided by the established involvement of AURKA–MYCN signaling in aggressive variant and neuroendocrine prostate cancer, the clinical evaluation of alisertib in CRPC/NEPC, and the role of enhancer of zeste homolog 2 (EZH2) in CRPC progression and neuroendocrine-associated epigenetic reprogramming [16,17,43,44,45,46,54,55,56,57].

**Figure 6:**
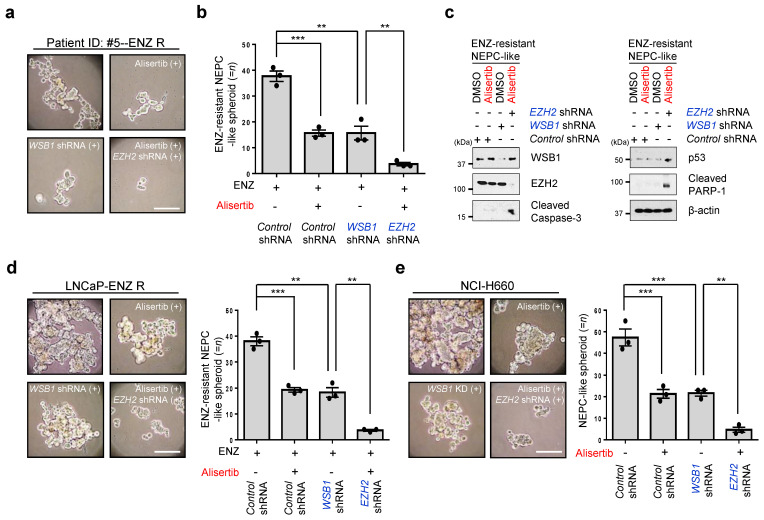
**Pharmacological and genetic perturbation of AURKA, *WSB1*, and *EZH2* reduces aggregate growth in established enzalutamide-resistant and neuroendocrine-like prostate cancer models.** (**a**) Representative bright-field images of Patient ID #5-derived enzalutamide (ENZ)-resistant spheroid-derived aggregates following control shRNA plus vehicle, control shRNA plus alisertib, *WSB1* shRNA plus vehicle, or *EZH2* shRNA plus alisertib treatment. Alisertib was used at 100 nM for 7 days. (**b**) Quantification of Patient ID #5-derived ENZ-resistant aggregates after the indicated treatments. (**c**) Immunoblot analysis of WSB1, EZH2, cleaved caspase-3, p53, cleaved poly(ADP-ribose) polymerase 1 (PARP-1), and β-actin in Patient ID #5-derived ENZ-resistant aggregates. β-actin was used as a loading control. Cleaved caspase-3 and cleaved PARP-1 were used as apoptosis-associated markers, whereas p53 was assessed as a stress-response and tumor suppressor pathway marker. (**d**) Representative images and quantification of LNCaP-derived ENZ-resistant aggregates after the indicated treatments. (**e**) Representative images and quantification of NCI-H660-derived neuroendocrine prostate cancer-like aggregates after the indicated treatments. Control shRNA indicates non-targeting short hairpin RNA; *WSB1* shRNA and *EZH2* shRNA indicate WSB1-targeting and EZH2-targeting short hairpin RNA, respectively. Quantitative data are shown as mean ± SEM from n = 3 independent experiments. Statistical significance in panels (**b**,**d**,**e**) was determined by one-way analysis of variance followed by Tukey’s multiple-comparisons test. ** *p* < 0.01, and *** *p* < 0.001. Scale bars, 50 µm.

In Patient ID #5-derived enzalutamide-resistant cultures, alisertib reduced resistant aggregate formation, and *WSB1* depletion produced a comparable inhibitory effect (Figure 6a,b). *EZH2* suppression combined with alisertib further decreased aggregate number, producing the strongest reduction among the tested conditions (Figure 6a,b). Immunoblot analysis showed altered WSB1 and EZH2 expression and increased apoptosis-associated markers, including cleaved caspase-3 and cleaved PARP-1, under selected treatment conditions, while p53 was assessed as a stress-response and tumor suppressor pathway marker (Figure 6c). These results suggest that the patient-derived enzalutamide-resistant NEPC-like state remains vulnerable to disruption of AURKA-sensitive signaling, WSB1-dependent activity, and EZH2-associated survival programs. We then evaluated whether this therapeutic pattern extended beyond the Patient ID #5-derived model. In LNCaP-derived enzalutamide-resistant cultures, alisertib, *WSB1* depletion, and combined alisertib/EZH2 suppression reduced resistant aggregate growth (Figure 6d). Similar inhibitory effects were observed in NCI-H660-derived neuroendocrine-like aggregates (Figure 6e). The concordant reduction in intact aggregate burden across the patient-derived, LNCaP-derived, and NCI-H660 models, together with induction of cleaved caspase-3 and cleaved PARP-1 in Patient ID #5-derived cultures, supports growth-inhibitory and apoptosis-associated effects following disruption of AURKA-, WSB1-, and EZH2-associated programs. Collectively, these data indicate that enzalutamide-resistant and NEPC-like prostate cancer spheroid models are supported by cooperating kinase-, ubiquitin-proteasome-, and chromatin-associated programs. Targeting these convergent dependencies may provide a rational strategy to constrain refractory NEPC-like growth without implying that WSB1 alone fully defines this disease state.

## 3. Discussion

This study establishes a biopsy-derived 3D spheroid platform for resolving how early prostate cancer adapts to sustained androgen receptor pathway inhibition and progresses toward a drug-resistant, neuroendocrine-like state. MRI-guided biopsy tissues generated viable patient-derived spheroids, and prolonged enzalutamide exposure selected a rare resistant population characterized by loss of prostate-lineage markers, induction of neuroendocrine-associated proteins, increased WSB1, and reduced PSMA. Importantly, the data define two temporally distinct intervention settings: *WSB1* depletion during early enzalutamide adaptation limited subsequent progression toward a resistant PSMA-low phenotype, whereas perturbation of AURKA-, WSB1-, and EZH2-associated programs reduced the growth of fully established refractory cultures. Together, these findings support WSB1-dependent PSMA ubiquitination as a candidate post-translational mechanism linking therapeutic adaptation to neuroendocrine-like progression.

A distinctive feature of this work is the longitudinal use of patient-derived spheroids established from MRI-guided biopsy specimens of early-stage prostate cancer to model early adaptation to sustained enzalutamide pressure, rather than relying exclusively on established cell lines or advanced tumor specimens. Conventional prostate cancer models have been indispensable for defining androgen receptor signaling and therapy resistance, but they incompletely represent the heterogeneity, tissue organization, and patient-specific biology of newly diagnosed disease. Patient-derived organoids and spheroids have begun to address this limitation, particularly in advanced prostate cancer and rare histologic subtypes, yet durable ex vivo culture from small diagnostic biopsy specimens remains technically difficult [29,30,31,32,33,34,35]. In this context, successful spheroid generation from 8 of 10 MRI-guided biopsy specimens provides more than a technical platform; it creates an experimental window into early adaptive events that are usually inaccessible until after CRPC or t-NEPC has already emerged clinically.

The emergence of an enzalutamide-resistant, t-NEPC-like derivative from a single patient-derived culture should be interpreted cautiously but is biologically informative. De novo NEPC is rare, accounting for less than 2% of prostate cancer at diagnosis, whereas treatment-emergent neuroendocrine or small-cell transformation has been reported in approximately 10–20% of metastatic CRPC cohorts exposed to potent androgen receptor pathway suppression [10,11,12,19,58,59]. These tumors are clinically aggressive, frequently androgen receptor-indifferent, often associated with low PSA relative to tumor burden, and poorly controlled by standard androgen receptor-directed therapy [10,11,12,13,14,15,16,17,18,19,58,59]. Thus, the rarity of this transition in our model is consistent with the idea that only a subset of early prostate tumors contains sufficient lineage plasticity or pre-existing drug-tolerant potential to undergo neuroendocrine-like remodeling. Our model does not imply that all early prostate cancers follow this path; instead, it provides a tractable system to study a clinically consequential but difficult-to-capture evolutionary route.

The PSMA component of this mechanism has direct clinical relevance. PSMA is highly expressed in most prostate adenocarcinomas and has become a central theranostic target for PET imaging and radioligand therapy [20,21,22,23,24,25,26,27]. Nevertheless, both PSMA expression and PSMA-ligand uptake can be heterogeneous, and low or negative signal in selected lesions may limit PSMA-based detection or eligibility for radioligand therapy [60,61,62]. In advanced disease, low PSMA expression is associated with poor outcomes in PSMA-directed treatment settings [27]. Neuroendocrine differentiation adds another layer of complexity because it is frequently associated with PSMA suppression [28]. Therefore, PSMA loss during enzalutamide-induced neuroendocrine-like progression represents not only a lineage change but also the potential loss of a diagnostic and therapeutic entry point. Restoring or preserving PSMA may create a second therapeutic opportunity for tumors that would otherwise become less visible and less targetable.

Consistent with prior evidence that WSB1 regulates malignant adaptation and metastatic progression through ubiquitin-dependent proteostasis [41,42,63,64,65], the present study provides convergent temporal, loss-of-function, cross-model, and reconstitution evidence linking WSB1 to the emergence of a PSMA-low resistant state. WSB1 increased as PSMA declined during enzalutamide selection (Figure 2d), and *WSB1* depletion initiated at week 6, when WSB1 induction first became evident, reduced subsequent resistant aggregate formation and restored PSMA expression at week 12 in Patient ID #5-derived spheroids (Figure 3a–c). *WSB1* silencing also reduced spheroid or aggregate growth in enzalutamide-resistant LNCaP, 22Rv1, and NCI-H660 models (Figure 3d–f), indicating that the observed dependency was not confined to a single patient-derived culture. Conversely, WSB1 reconstitution in LNCaP cells reinstated a PSMA-low, AR-low, CgA-high molecular profile (Figure 4a), supporting an on-target and reversible WSB1-dependent phenotype. Together, these findings position WSB1 as a functional contributor to progression from early enzalutamide adaptation toward a resistant PSMA-low state, rather than as a passive marker of lineage remodeling. Biochemically, the data place PSMA within a WSB1-dependent ubiquitination pathway. Wild-type WSB1 increased high-molecular-weight ubiquitinated PSMA species, whereas deletion of the SOCS box strongly reduced this activity (Figure 4c), indicating that recruitment of the E3 ligase machinery is required for efficient PSMA ubiquitination. The T380A mutant also displayed reduced activity toward PSMA, and alisertib attenuated WSB1-associated PSMA ubiquitination under the tested conditions (Figure 5c). These findings support a model in which the SOCS box provides the essential ubiquitin-ligase adaptor function, while the conserved T380 region and AURKA-sensitive signaling modulate WSB1 activity toward PSMA. However, the current data do not establish that AURKA directly phosphorylates WSB1 at T380, which was previously linked to CDK-dependent phosphorylation [42]. Likewise, endogenous WSB1–PSMA interaction, PSMA protein half-life, proteasome-dependent rescue, and ubiquitin-linkage analyses will be required to establish PSMA as a bona fide WSB1 substrate.

**Figure 7:**
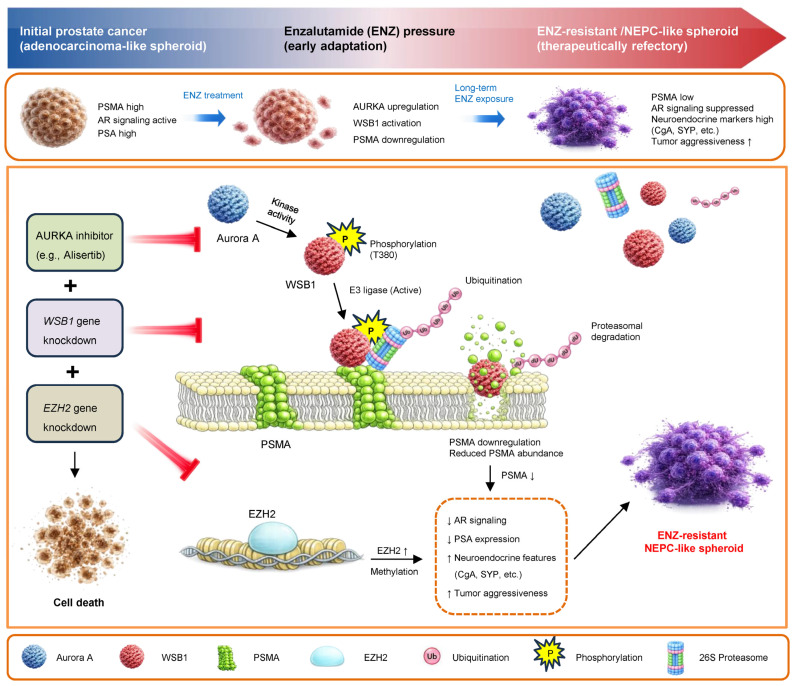
**Proposed model of the AURKA–WSB1–PSMA axis in enzalutamide-induced neuroendocrine-like progression of prostate cancer.** Early prostate cancer spheroids maintain an adenocarcinoma-like phenotype with high PSMA expression, active AR signaling, and high PSA expression. Sustained ENZ pressure promotes adaptive resistance and drives a subset of spheroids toward an NEPC-like refractory state. Mechanistically, AURKA-sensitive signaling is proposed to modulate WSB1 activity, potentially through the conserved T380 region, whereas the SOCS box provides the E3 ligase adaptor module required for PSMA ubiquitination. Activated WSB1 promotes PSMA ubiquitination and proteasomal degradation, leading to reduced PSMA abundance, reduced AR signaling and PSA expression, and increased neuroendocrine markers, including CgA and SYP. Pharmacologic AURKA inhibition, *WSB1* depletion, or *EZH2* suppression may interrupt this transition by limiting PSMA degradation and reducing survival of ENZ-resistant NEPC-like spheroids. Ub, ubiquitin; P, phosphorylation.

Current treatment options for NEPC and aggressive-variant prostate cancer remain limited; platinum-based chemotherapy is commonly used, but responses are often transient and durable targeted strategies are lacking [10,11,12,13,14,15,16,17,18,19,58,59]. Against this background, the intervention studies distinguish prevention of resistant progression from treatment of an established refractory state. In Figure 3, *WSB1* depletion initiated during early enzalutamide adaptation limited subsequent development of the PSMA-low resistant phenotype. By contrast, the experiments in Figure 6 were performed after enzalutamide-resistant cultures had been established and therefore tested whether the refractory state remained therapeutically vulnerable. Under these conditions, AURKA inhibition with alisertib, *WSB1* suppression, and *EZH2* depletion each reduced resistant spheroid or aggregate growth, while combined alisertib treatment with *EZH2* suppression produced the greatest reduction among the tested conditions without establishing formal pharmacologic synergy. This stage-specific vulnerability is consistent with the established involvement of AURKA–MYCN signaling and EZH2-associated epigenetic reprogramming in aggressive-variant and neuroendocrine prostate cancer [16,17,43,44,45,46,54,55,56,57]. Rather than identifying WSB1 as a solitary determinant, the data support a model in which AURKA-sensitive signaling, WSB1-dependent proteolysis, and EZH2-associated chromatin maintenance constitute cooperating dependencies of the refractory state. Figure 7 summarizes this framework. Intercepting WSB1 during early adaptation may constrain resistant progression, whereas targeting these convergent dependencies after resistance has emerged may reduce resistant aggregate growth and increase apoptosis-associated signaling in established NEPC-like cultures.

Several limitations should be considered. First, the strongest patient-derived resistant phenotype was observed in Patient ID #5, limiting generalizability. Because t-NEPC is heterogeneous and relatively uncommon even in metastatic CRPC cohorts, this single patient-derived transition model should be viewed as a discovery platform rather than a prevalence estimate [10,11,12,19,58,59]. Second, the resistant phenotype should remain designated as NEPC-like or t-NEPC-like rather than definitive t-NEPC. CgA/SYP induction with AR/PSA/PSMA loss provides a strong neuroendocrine-like signature, but definitive classification requires broader pathologic validation, including insulinoma-associated protein 1, CD56/neural cell adhesion molecule 1, achaete-scute family basic helix-loop-helix transcription factor 1, neuron-specific enolase, NKX3.1 loss, and histologic evaluation [59,66]. Additional MYCN, AURKA, and EZH2 profiling, together with transcriptomic comparison to clinical t-NEPC, would further strengthen lineage-state assignment [16,17,43,54,55,56,57]. Additional biopsy-derived models linked to clinical pathology, genomic profiling, and treatment history will be essential to determine whether WSB1 induction is recurrent or patient-specific. Third, the therapeutic perturbation studies evaluated spheroid or aggregate burden and apoptosis-associated protein cleavage but did not include a direct post-treatment metabolic or fluorescence-based viability assay. Although the concordant reduction in aggregate growth across three prostate cancer models and the induction of cleaved caspase-3 and cleaved PARP-1 support growth-inhibitory and pro-apoptotic effects, these endpoints do not constitute a quantitative measurement of absolute cell viability or cytotoxic potency. Accordingly, the present findings should be interpreted as evidence of reduced resistant aggregate growth and apoptosis-associated signaling rather than as a direct viability endpoint.

Additional mechanistic and translational validation could address three points. First, *FOLH1* transcription, total and surface PSMA abundance, protein turnover, ligand-binding capacity, and radioligand uptake should be evaluated in parallel to distinguish transcriptional repression from post-translational degradation and to determine whether restored PSMA is functionally targetable [28,54,55,56,57]. Second, the AURKA–WSB1 relationship requires orthogonal genetic and biochemical validation, including AURKA perturbation, kinase-dead AURKA and Aurora kinase B controls, phospho-WSB1 mapping, additional AURKA-selective inhibitors, and in vitro kinase analysis. Third, future therapeutic studies should incorporate direct three-dimensional metabolic or fluorescence-based viability assays with appropriate biological replication, together with dose–response analysis, treatment-withdrawal regrowth assays, and formal combination testing. Future animal studies should then evaluate the two temporally distinct intervention settings identified in the present study. In an early-intervention model, PSMA-high LNCaP or patient-derived adenocarcinoma-like spheroids would be implanted into male immunodeficient mice, followed by androgen deprivation and continuous enzalutamide exposure after tumor establishment. Inducible *WSB1* depletion would be initiated after early WSB1 induction and PSMA decline but before overt resistance to determine whether WSB1 suppression delays progression toward an enzalutamide-resistant, NEPC-like state. In an established-resistance model, LNCaP-derived enzalutamide-resistant and NCI-H660 xenografts, together with Patient ID #5-derived resistant spheroid xenografts if sufficient viable material and engraftment are achieved, would be randomized to vehicle, alisertib, inducible *WSB1* depletion, *EZH2* inhibition, or combined AURKA/EZH2 targeting. Longitudinal tumor growth and time to progression would be integrated with serial PSMA PET imaging and endpoint analyses of WSB1, PSMA, AR, CgA, SYP, Ki67, and cleaved caspase-3. If pathway inhibition restores tumor PSMA uptake, subsequent treatment with ^177^Lu-PSMA-617 would determine whether the restored PSMA is functionally accessible for radioligand therapy [23,24,25,26]. Complementary Pten/Rb1/Trp53- or MYCN/EZH2-based genetically engineered models could subsequently evaluate WSB1-dependent lineage plasticity in an immunocompetent microenvironment [13,14,16,17]. These studies will determine whether the pathway identified here represents a reproducible therapeutic dependency rather than a context-specific feature of the current models.

Overall, these findings define a stage-resolved model in which WSB1 is induced during early enzalutamide adaptation, promotes PSMA loss through a SOCS box-dependent ubiquitination program, and remains embedded within a therapeutically actionable signaling network after resistance is established. Validation in larger patient-derived cohorts and in vivo models will determine whether targeting this network can delay neuroendocrine-like progression or restore PSMA-directed theranostic sensitivity.

## 4. Materials and Methods

### 4.1. Human Prostate Cancer Biopsy Specimens and Ethical Approval

Human prostate biopsy specimens were obtained at Mayo Clinic under an Institutional Review Board-approved protocol (Mayo Clinic IRB 24-006965; approved 30 October 2024). Patient enrollment began on 18 April 2025. Written informed consent was obtained from all participants before research tissue collection. Study procedures were conducted in accordance with the Declaration of Helsinki and approved institutional guidelines. A total of 14 patients undergoing clinically indicated magnetic resonance imaging (MRI)-guided prostate biopsy for suspected or diagnosed early-stage prostate cancer were enrolled. Of these, research biopsy specimens from 10 patients were ultimately available and included in the present study for three-dimensional (3D) spheroid establishment and downstream experimental analyses. Specimens from four enrolled patients were not included because the clinical evaluation identified normal findings in some cases, biopsy scheduling was postponed in some cases, or an additional research core could not be provided because of limited biopsy core availability. Biopsy procedures were performed as part of standard clinical care using in-bore MRI-guided biopsy within a combined targeted/systematic biopsy approach. Research specimens were obtained from imaging-defined areas of clinical suspicion. After completion of clinically required diagnostic sampling, an additional research biopsy core was collected when feasible and without compromising clinical diagnostic evaluation. Because viable tissue was required for ex vivo culture, research specimens were transferred for experimental processing immediately after collection. Patient-related information was de-identified before laboratory processing, and all specimens were assigned coded study identifiers. Fresh biopsy tissues were collected in collaboration with the clinical MRI-guided biopsy team and transferred immediately for downstream experimental processing.

### 4.2. Biopsy Tissue Processing, Growth Factor-Enriched 3D Spheroid Culture, Morphologic Analysis, and Long-Term Enzalutamide Selection

Fresh prostate biopsy tissues allocated for research were transferred immediately after collection into pre-chilled sterile transport medium and maintained on ice until processing. Samples were transported to the laboratory at 4 °C and processed as rapidly as possible to minimize ischemic injury. Under sterile conditions, tissues were washed three times with cold phosphate-buffered saline (PBS) supplemented with 1% penicillin-streptomycin to remove blood, mucus, and tissue debris. Because MRI-guided prostate biopsy cores contain limited viable tumor material and are highly vulnerable to ischemic and mechanical stress, all procedures were performed gently to preserve multicellular tissue fragments, epithelial/tumor-cell clusters, and native cell–cell interactions. Biopsy tissues were mechanically fragmented on ice into approximately 0.5–1.0 mm tissue pieces using sterile microdissection instruments. Complete single-cell dissociation was intentionally avoided. To release compact epithelial/tumor clusters while minimizing overdigestion, tissue fragments were subjected to mild enzymatic digestion in Advanced DMEM/F12 (Gibco, Thermo Fisher Scientific, Waltham, MA, USA) containing Collagenase/Hyaluronidase (STEMCELL Technologies, Vancouver, BC, Canada; 1:10 dilution from stock), DNase I (Sigma-Aldrich, St. Louis, MO, USA; 20 μg/mL), and Y-27632 (Selleck Chemicals, Houston, TX, USA; 10 μM) for 20–30 min at 37 °C with gentle rocking. Digestion was terminated by adding excess cold spheroid culture medium, and tissue fragments were collected by low-speed centrifugation at 300× *g* for 5 min at 4 °C. The pellet was gently resuspended without vigorous pipetting to retain multicellular fragments and tumor-cell clusters. Processed fragments were seeded into 6-well 3D culture plates using either Nunclon^TM^ Sphera^TM^ Low-Attachment Multidishes (Thermo Fisher Scientific, Waltham, MA, USA; Cat. No. 174932) or NanoCulture plates (SCIVAX Corporation, Kawasaki, Kanagawa, Japan). Plate type was recorded for each culture, and all within-experiment comparisons were performed using the same 3D plate format to minimize plate-dependent differences in spheroid formation.

Primary cultures were maintained in serum-free, growth factor-enriched prostate spheroid medium adapted from established prostate organoid culture systems, with modifications for low-attachment biopsy-derived spheroid culture rather than long-term extracellular matrix-embedded organoid expansion [29,30,31,32,33]. The basal medium consisted of Advanced DMEM/F12 supplemented with 1× B27 supplement, 1.25 mM of N-acetyl-L-cysteine (Sigma-Aldrich, St. Louis, MO, USA), 10 mM of HEPES (Gibco, Waltham, MA, USA), 2 mM of GlutaMAX (Thermo Fisher Scientific, Waltham, MA, USA), and 1% penicillin-streptomycin (Gibco, Waltham, MA, USA). To support prostate epithelial survival, androgen-lineage maintenance, and recovery from biopsy-associated stress, the medium was supplemented with epidermal growth factor (EGF; 50 ng/mL; PeproTech, Cranbury, NJ, USA), fibroblast growth factor 10 (FGF10; 10 ng/mL; PeproTech), fibroblast growth factor 2 (FGF2; 5 ng/mL; PeproTech), dihydrotestosterone (DHT; 1 nM; Sigma-Aldrich), nicotinamide (10 mM; Sigma-Aldrich), A83-01 (500 nM; Tocris Bioscience, Abingdon, UK), SB202190 (10 μM; Sigma-Aldrich), Y-27632, an inhibitor of Rho-associated coiled-coil-containing protein kinases 1 and 2 (ROCK1/2) (10 μM; Selleck Chemicals), prostaglandin E2 (PGE2; 1 μM; Tocris Bioscience), recombinant R-spondin 1 (500 ng/mL; R&D Systems, Minneapolis, MN, USA), and recombinant Noggin (100 ng/mL; R&D Systems, Minneapolis, MN, USA). The medium also contained A83-01, an inhibitor of transforming growth factor-β type I receptor/ALK5 and the related ALK4 and ALK7 kinases (500 nM; Tocris Bioscience); SB202190, a p38 mitogen-activated protein kinase inhibitor (10 μM; Sigma-Aldrich); and Y-27632, a ROCK1/2 inhibitor (10 μM; Selleck Chemicals). This cocktail was selected based on prostate organoid media that support prostate epithelial and prostate cancer organoid growth through EGF/FGF signaling, Wnt potentiation, bone morphogenetic protein inhibition, transforming growth factor-β/activin receptor signaling inhibition, p38 mitogen-activated protein kinase inhibition, ROCK1/2 inhibition, and androgen pathway support [29,30,31,32,33]. Cultures were maintained in a final volume of 4.0 mL per well. At each medium exchange, 3.5 mL of fresh growth factor-enriched spheroid medium was added together with 0.5 mL of retained autologous conditioned medium from the same well to preserve locally secreted autocrine and paracrine factors while allowing nutrient replenishment. Cultures were maintained at 37 °C in a humidified incubator containing 5% CO_2_, and medium was refreshed every 3–5 days using the same conditioned-medium retention strategy.

Spheroid formation was monitored for up to 4 weeks by bright-field and phase-contrast microscopy with an EVOS M5000 imaging system (Thermo Fisher Scientific, Waltham, MA, USA). Countable tumor spheroids were operationally defined as viable-appearing, compact, rounded or near-rounded multicellular aggregates with clear outer borders and a minimum diameter of 50 μm. Single cells, blood clots, acellular debris, necrotic fragments, and loose non-compact clusters were excluded from analysis. Tumor relevance and lineage state were supported by pathology confirmation of corresponding MRI-targeted diagnostic cores and by downstream marker analysis. Parental adenocarcinoma-like spheroids were evaluated using prostate lineage markers, including androgen receptor (AR), prostate-specific antigen (PSA), and prostate-specific membrane antigen (PSMA), whereas enzalutamide-resistant neuroendocrine-like derivatives were evaluated using chromogranin A (CgA), synaptophysin (SYP), AR, PSA, PSMA, and WSB1. For spheroid quantification, conditioned medium was gently mixed to resuspend floating spheroids without disrupting compact aggregates, and a standardized 0.5 mL aliquot was collected at each indicated time point. Spheroid numbers were determined by manual counting and/or image-analysis software. Where applicable, spheroid area, equivalent diameter, circularity, solidity, and boundary roughness were quantified using ImageJ version 1.54g (National Institutes of Health, Bethesda, MD, USA) or equivalent software. Equivalent circular diameter was calculated as 2 × √(area/π). For long-term drug selection, established patient-derived spheroid cultures were subjected to continuous enzalutamide (Ambeed, Inc., Arlington Heights, IL, USA; Cat. No. A270395; CAS No. 915087-33-1) treatment at a final concentration of 10 μM for up to 12 weeks. Vehicle controls were maintained with the equivalent dimethyl sulfoxide (DMSO; Sigma-Aldrich, St. Louis, MO, USA; Cat. No. D2650) concentration, with a final concentration of approximately 0.01%. Samples were collected at week 0, week 6, and week 12 for molecular analysis. Cultures that failed to maintain viable spheroid growth under enzalutamide were classified as enzalutamide-sensitive, whereas cultures that persisted and expanded under continuous drug pressure were classified as enzalutamide-resistant spheroid-derived aggregates. Scale bars were generated using microscope-calibrated pixel-to-micrometer conversion factors and preserved during image processing.

### 4.3. Tumor-Spheroid Viability Assay

During culture week 4, Patient ID #5-derived spheroids cultured under the three-dimensional conditions described above were treated with vehicle (dimethyl sulfoxide [DMSO]) or paclitaxel (Sigma-Aldrich, St. Louis, MO, USA; Cat. No. T7191) at a final concentration of 10 nM for 48 h. Vehicle-treated cultures received DMSO at the same final concentration used for paclitaxel treatment. Following treatment, spheroids were gently collected and washed twice with phosphate-buffered saline (PBS). Spheroid viability was assessed using the LIVE/DEAD^TM^ Cell Viability Assay (Invitrogen, Thermo Fisher Scientific, Waltham, MA, USA; Cat. No. L3224) according to the manufacturer’s instructions. Spheroids were incubated in staining solution containing 2 μM of calcein-AM and 4 μM of ethidium homodimer-1 (EthD-1) for 45 min at room temperature in the dark. After staining, the spheroids were washed twice with PBS and immediately examined by bright-field using an EVOS M5000 Imaging System (Thermo Fisher Scientific, Waltham, MA, USA) and by confocal fluorescence microscopy using an LSM 980 confocal microscope equipped with Airyscan 2 (Carl Zeiss Microscopy GmbH, Jena, Germany). Confocal fluorescence images of vehicle- and paclitaxel-treated spheroids were acquired using ZEN software, version 3.6 (Carl Zeiss Microscopy GmbH), with identical laser power, detector gain, and acquisition settings across all experimental groups. Calcein-AM fluorescence was used to identify viable cells, whereas EthD-1 fluorescence was used to identify dead cells with compromised plasma membranes. Vehicle-treated spheroids were used to assess baseline viability, whereas paclitaxel-treated spheroids served as a positive control for cell death.

### 4.4. Prostate Cancer Cell Lines and Cell Line-Derived 3D Spheroid Models

Human prostate cancer cell lines were obtained from the American Type Culture Collection (ATCC, Manassas, VA, USA) and maintained according to ATCC recommendations and established literature protocols [47,48,49,50]. LNCaP cells (ATCC CRL-1740), an androgen receptor-positive prostate adenocarcinoma model, were cultured in RPMI-1640 medium (Gibco, Thermo Fisher Scientific, Waltham, MA, USA) supplemented with 10% fetal bovine serum (FBS; Gibco), 100 U/mL of penicillin, and 100 μg/mL of streptomycin (Gibco) [47]. VCaP cells (ATCC CRL-2876), used as an additional prostate adenocarcinoma reference model, were maintained in Dulbecco’s Modified Eagle Medium (DMEM; Gibco) supplemented with 10% FBS, 100 U/mL of penicillin, and 100 μg/mL of streptomycin [48]. 22Rv1 cells (ATCC CRL-2505), used as an androgen receptor-positive castration-resistant prostate cancer-like reference model, were cultured in RPMI-1640 medium supplemented with 10% FBS, 100 U/mL of penicillin, and 100 μg/mL of streptomycin [49]. NCI-H660 cells (ATCC CRL-5813), used as a neuroendocrine/small-cell prostate carcinoma reference model, were maintained in HITES medium supplemented with 5% FBS, insulin (5 μg/mL; Sigma-Aldrich, St. Louis, MO, USA; Cat. No. I9278), transferrin (10 μg/mL; Sigma-Aldrich; Cat. No. T8158), sodium selenite (30 nM; Sigma-Aldrich; Cat. No. S5261), hydrocortisone (10 nM; Sigma-Aldrich; Cat. No. H0888), β-estradiol (10 nM; Sigma-Aldrich; Cat. No. E2758), additional L-glutamine to a final concentration of 4 mM (Gibco, Thermo Fisher Scientific, Waltham, MA, USA; Cat. No. 25030081), 100 U/mL of penicillin, and 100 μg/mL of streptomycin (Gibco, Thermo Fisher Scientific; Cat. No. 15140122) [50]. NCI-H660 cultures were maintained as viable floating clusters and were not over-diluted during passaging, consistent with their aggregate-forming growth pattern. All cell cultures were maintained at 37 °C in a humidified incubator containing 5% CO_2_ and were passaged before reaching overconfluence or excessive acidification of the culture medium. Cells were periodically renewed from authenticated frozen master stocks, and cultures were routinely screened for mycoplasma contamination; only mycoplasma-negative cultures were used for experiments.

For cell line-derived 3D culture experiments, LNCaP, VCaP, 22Rv1, and NCI-H660 cells were adapted to spheroid or aggregate culture using the same 3D culture plate formats used for patient-derived spheroid experiments, including Nunclon^TM^ Sphera^TM^ Low-Attachment Multidishes or NanoCulture plates. LNCaP and VCaP cells were used as prostate adenocarcinoma spheroid controls, 22Rv1 cells were used to model a CRPC-like androgen receptor-positive state, and NCI-H660 cells were used as a neuroendocrine/small-cell prostate carcinoma aggregate reference. For 3D assays, cells were harvested under conditions that preserved viability and minimized excessive single-cell stress, counted, and seeded into 6-well 3D culture plates in their corresponding complete culture media unless otherwise indicated. LNCaP-derived enzalutamide-resistant cultures were generated by long-term enzalutamide exposure, using the same selection strategy applied to patient-derived spheroids. Cell line-derived spheroids or aggregates were monitored by bright-field microscopy and analyzed by spheroid/aggregate counting, morphologic assessment, and immunoblotting. For within-experiment comparisons, the same cell line, medium condition, plate format, seeding density, and drug-treatment schedule were used across experimental groups to minimize culture-condition-dependent variability.

### 4.5. Gene Knockdown and Plasmid Expression

*WSB1* and *EZH2* expressions were suppressed using short hairpin RNA (shRNA)-based gene silencing. Non-targeting shRNA in the corresponding vector backbone was used as a negative control. Human *WSB1*-targeting shRNA constructs were obtained from Open Biosystems (Huntsville, AL, USA) and have been described previously [41,42]. Myc-tagged WSB1 expression constructs were also described previously [41,42]. Two independent human *WSB1* shRNA constructs were used where indicated: *WSB1* shRNA #1, 5′-GCTGTTGACAGTGAGCGCGGAGTTTCTCTCGTATCGTATTAGTGAAGCCACAGATGTAATACGATACGAGAGAAACTCCATGCCTACTGCCTCGGA-3′; and *WSB1* shRNA #2, 5′-GCTGTTGACAGTGAGCGCGCTGTAAAGTGCAAGGAAATTTAGTGAAGCCACAGATGTAAATTTCCTTGCACTTTACAGCATGCCTACTGCCTCGGA-3′ [41,42]. *EZH2* was depleted using the following human *EZH2*-targeting shRNA sequence: 5′-CCGGTATGATGGTTAACGGTGATCACTCGAGTGATCACCGTTAACCATCATATTTTTG-3′. For stable *WSB1* or *EZH2* suppression, lentiviral particles were generated in HEK293T packaging cells by co-transfecting the indicated shRNA transfer plasmids with lentiviral packaging and envelope plasmids using Lipofectamine^TM^ 2000 or Lipofectamine^TM^ 3000 (Invitrogen, Thermo Fisher Scientific, Waltham, MA, USA). Viral supernatants were collected, clarified by centrifugation and filtration, and applied to prostate cancer cells or spheroid-derived cultures under aseptic conditions. For *WSB1* knockdown experiments, the non-targeting control and *WSB1*-targeting shRNAs were expressed from the same puromycin-selectable vector backbone, and both groups underwent identical lentiviral transduction and antibiotic-selection procedures. For the experiments examining WSB1 function during enzalutamide adaptation, Patient ID #5-derived cultures were transduced at week 6 of continuous enzalutamide exposure, when WSB1 induction was first detected in the time-course analysis. Parallel vehicle-treated cultures were transduced at the corresponding time point. Following transduction, cultures were selected with puromycin (InvivoGen, San Diego, CA, USA; Cat. Code ant-pr-1) at a final concentration of 2 μg/mL until stable puromycin-resistant pools were established. The selected cultures were maintained under their respective vehicle or continuous enzalutamide conditions and analyzed at week 12, corresponding to six weeks after lentiviral transduction. The timing of genetic perturbation in fully established enzalutamide-resistant cultures used for therapeutic studies is described in Section 4.8.

For rescue and gain-of-function experiments, empty Myc vector, Myc-tagged WSB1 wild type, Myc-tagged WSB1 ΔSOCS, and Myc-tagged WSB1 T380A constructs were introduced as indicated. Myc-WSB1 ΔSOCS was used to assess the requirement of the SOCS box region, whereas Myc-WSB1 T380A was used to evaluate the contribution of the conserved T380 residue. For ubiquitination assays, His-tagged ubiquitin was co-expressed with the indicated WSB1 constructs. For transient plasmid expression in LNCaP cells, DNA constructs were introduced using Lipofectamine^TM^ 2000 or Lipofectamine^TM^ 3000 (Invitrogen/Thermo Fisher Scientific) according to the manufacturer’s instructions. Transfection conditions were empirically optimized to achieve reproducible gene delivery with minimal cytotoxicity. Mock-transfected and vehicle-only controls were included where appropriate to monitor reagent-related effects. Transfection efficiency was verified by fluorescence microscopy when reporter-tagged constructs were used and by immunoblotting for target protein expression. Transiently transfected cells were typically analyzed 48–72 h after transfection. The efficiency of WSB1 depletion, EZH2 suppression, and ectopic WSB1 reconstitution was confirmed by immunoblotting for WSB1, EZH2, and Myc-tag, respectively. PSMA, AR, and CgA were analyzed as downstream molecular markers, and β-actin was used as a loading control, as appropriate.

### 4.6. Immunoblotting and Antibodies

Spheroids, spheroid-derived aggregates, and cultured prostate cancer cells were collected at the indicated time points and washed twice with ice-cold phosphate-buffered saline (PBS). Spheroid and aggregate cultures were collected by gentle, low-speed centrifugation to minimize mechanical disruption. Total protein lysates were prepared in radioimmunoprecipitation assay (RIPA) buffer containing 50 mM Tris-HCl, pH 7.4, 150 mM NaCl, 1% NP-40/Igepal CA-630, 0.5% sodium deoxycholate, and 0.1% sodium dodecyl sulfate (SDS), freshly supplemented with cOmplete^TM^ Mini, EDTA-free Protease Inhibitor Cocktail (Roche Diagnostics GmbH, Mannheim, Germany; Cat. No. 11836170001) and phosphatase inhibitors consisting of 10 mM β-glycerophosphate, 10 mM sodium fluoride, and 1 mM sodium orthovanadate. Lysates were incubated on ice for 20–30 min with intermittent mixing and clarified by centrifugation at 12,000–14,000× *g* for 10–15 min at 4 °C. Protein concentrations were determined using the Bradford assay with Bio-Rad Protein Assay Dye Reagent Concentrate (Bio-Rad Laboratories, Hercules, CA, USA; Cat. No. 5000006). Immunoblotting procedures were adapted from previously described WSB1 studies, with modifications for prostate cancer spheroid and cell-line lysates [41,42]. Equal amounts of protein, typically 10–30 μg per lane depending on sample availability, were mixed with SDS sample buffer containing β-mercaptoethanol, denatured at 97 °C for 5 min, and immediately chilled on ice. Proteins were resolved by SDS-polyacrylamide gel electrophoresis using 4–15% Mini-PROTEAN® TGX^TM^ Precast Protein Gels, 15-well, 15-μL format (Bio-Rad Laboratories; Cat. No. 4561086), at 80–120 V for 1–2 h in an ice-cooled electrophoresis tank. Proteins were transferred for 7 min onto 0.2-μm nitrocellulose membranes using a Trans-Blot® Turbo^TM^ RTA Mini 0.2-μm Nitrocellulose Transfer Kit (Bio-Rad Laboratories; Cat. No. 1704270) and a Trans-Blot Turbo semi-dry transfer system (Bio-Rad Laboratories; Cat. No. 1704150). Membranes were blocked with 5% nonfat dry milk for 1 h at room temperature, washed three times with Tris-buffered saline containing 0.1% Tween-20 (TBS-T), and incubated overnight at 4 °C with the indicated primary antibodies, typically diluted 1:500–1:1000. After three washes with TBS-T, the membranes were incubated with the appropriate HRP-conjugated secondary antibodies for 1 h at room temperature. Depending on the host species of the primary antibody, the secondary antibodies included goat anti-rabbit IgG H&L (HRP) (Abcam, Cambridge, UK; Cat. No. ab6721), goat anti-mouse IgG H&L (HRP) (Abcam; Cat. No. ab6789), anti-rabbit IgG HRP-linked antibody (Cell Signaling Technology, Danvers, MA, USA; Cat. No. 7074), or anti-mouse IgG HRP-linked antibody (Cell Signaling Technology; Cat. No. 7076). Chemiluminescent signals were developed using Pierce^TM^ ECL Western Blotting Substrate (Thermo Fisher Scientific, Waltham, MA, USA; Cat. No. 32209) and recorded under nonsaturating exposure conditions on PR1MA^TM^ Blue autoradiography and chemiluminescence film, 8 × 10 inches (Midwest Scientific, Fenton, MO, USA; Cat. No. BX810). When required, membranes were stripped and reprobed according to standard procedures. Band intensities were quantified by densitometry using ImageJ, version 1.54g (National Institutes of Health, Bethesda, MD, USA), and normalized to β-actin unless otherwise indicated. The following primary antibodies were used: WSB1 (Proteintech, Rosemont, IL, USA, #11666-1-AP; Abcam, Cambridge, UK, ab68953; Sigma, HPA003293), prostate-specific membrane antigen (PSMA; Thermo Fisher Scientific/Invitrogen, Waltham, MA, USA, #37-3900; Abcam, AB133579), androgen receptor (AR; Cell Signaling Technology, Danvers, MA, USA, #3202; Santa Cruz Biotechnology, sc-7305), prostate-specific antigen (PSA; Abcam, #ab53774; Abcam, AB76113), chromogranin A (CgA; Santa Cruz Biotechnology, sc-393941; Novus Biologicals, Centennial, CO, USA, NBP2-33198), synaptophysin (SYP; Cell Signaling Technology, #4329; Santa Cruz Biotechnology, sc-12737), EZH2 (Cell Signaling Technology, #5246; Proteintech, # 21800-1-AP), cleaved caspase-3 (Cell Signaling Technology, #9661; Abcam, AB32042), p53 (Abcam, ab131442; Cell Signaling Technology, #9282), cleaved poly(ADP-ribose) polymerase 1 (PARP-1; Cell Signaling Technology, #9541; ABclonal, Woburn, MA, USA, A19612), Myc-tag (Cell Signaling Technology, #3400), and β-actin (Cell Signaling Technology, #3700S). Primary antibodies were used at manufacturer-recommended or empirically optimized dilutions, typically ranging from 1:500 to 1:1000 for target proteins and 1:2000 to 1:5000 for β-actin. Horseradish peroxidase-conjugated anti-rabbit and anti-mouse secondary antibodies were used at 1:2000 to 1:5000 dilutions. β-actin served as the loading control for total lysates unless otherwise specified.

### 4.7. Ubiquitination Assay and Denaturing Ni-NTA Pull-Down

To determine whether WSB1 promotes PSMA ubiquitination, denaturing nickel-nitrilotriacetic acid (Ni-NTA) pull-down assays were performed in LNCaP cells. This assay was adapted from previously described WSB1 ubiquitination protocols with modifications for prostate cancer cells and PSMA detection [41,42]. LNCaP cells were seeded to approximately 60–70% confluence and co-transfected with His-tagged ubiquitin together with empty Myc vector, Myc-tagged WSB1 wild type, Myc-tagged WSB1 ΔSOCS, or Myc-tagged WSB1 T380A constructs, as indicated. Transfections were performed using Lipofectamine^TM^ 2000 or Lipofectamine^TM^ 3000 according to the manufacturer’s instructions. Where indicated, cells were treated with alisertib (MedChemExpress, Monmouth Junction, NJ, USA; Cat. No. HY-10971) before harvest to assess Aurora kinase A-sensitive regulation of WSB1-dependent PSMA ubiquitination. To stabilize ubiquitinated intermediates, cells were treated with MG132 (Sigma-Aldrich, St. Louis, MO, USA; Cat. No. M7449) at 10 μM for 4 h before collection. Cells were washed twice with ice-cold phosphate-buffered saline (PBS) and lysed under denaturing conditions to enrich covalently ubiquitinated proteins while disrupting non-covalent protein interactions. Denaturing lysis was performed in 6 M of guanidine-HCl buffer containing 0.1 M of Na_2_HPO_4_/NaH_2_PO_4_, 0.1 M of Tris-HCl, pH 8.0, 300 mM of NaCl, 10 mM of imidazole, and 0.05% Tween-20, freshly supplemented with 20 mM of N-ethylmaleimide and 5 mM of iodoacetamide (all from Sigma-Aldrich) to inhibit deubiquitination and preserve ubiquitin conjugates. Lysates were briefly sonicated to reduce viscosity and clarified by centrifugation at 14,000× *g* for 10 min at 4 °C. A fraction of each lysate was retained as input before pull-down. Clarified denatured lysates were incubated with Ni-NTA agarose beads (Qiagen, Hilden, Germany), pre-equilibrated in denaturing lysis buffer, for 4 h at room temperature with gentle rotation. Beads were washed sequentially with denaturing lysis buffer containing 20 mM of imidazole, followed by urea-based wash buffer containing 8 M of urea (Sigma-Aldrich), 0.1 M of Na_2_HPO_4_/NaH_2_PO_4_, 0.1 M of Tris-HCl, pH 6.3, 300 mM of NaCl, and 0.05% Tween-20 to reduce nonspecific binding. Bound His-ubiquitin-conjugated proteins were eluted by boiling the beads in 2× SDS sample buffer containing 200 mM of imidazole and 5% β-mercaptoethanol. Eluted proteins were resolved by SDS-polyacrylamide gel electrophoresis and analyzed by immunoblotting using anti-PSMA antibody to detect ubiquitinated PSMA species. Input lysates were analyzed in parallel for PSMA, Myc-tagged WSB1, and β-actin. Empty vector and non-His-ubiquitin controls were included where appropriate to assess nonspecific Ni-NTA binding and background signal. High-molecular-weight PSMA-reactive species in the Ni-NTA pull-down fraction were interpreted as ubiquitinated PSMA species.

### 4.8. Genetic and Pharmacological Perturbation of Established Enzalutamide-Resistant and Neuroendocrine-like Spheroid and Aggregate Cultures

Established enzalutamide-resistant and neuroendocrine-like prostate cancer spheroid and aggregate cultures were used for genetic and pharmacological perturbation studies. Patient ID #5-derived and LNCaP-derived enzalutamide-resistant cultures were first established by continuous enzalutamide selection for 12 weeks. After the resistant spheroid-derived aggregate phenotype had been established, cultures were transduced with lentiviruses encoding non-targeting control, *WSB1*-targeting, or *EZH2*-targeting shRNA and selected with puromycin at 2 μg/mL to generate stable shRNA-expressing pools. Patient ID #5-derived enzalutamide-resistant spheroid-derived aggregates and LNCaP-derived enzalutamide-resistant cultures were maintained under continuous enzalutamide pressure at 10 μM throughout treatment to preserve the drug-resistant state. NCI-H660 cells were used as a neuroendocrine/small-cell prostate carcinoma reference model and were maintained under their standard three-dimensional aggregate culture conditions without prior enzalutamide selection. Where indicated, stable control, *WSB1*-targeting, or *EZH2*-targeting shRNA-expressing NCI-H660 cultures were generated using the same lentiviral transduction and puromycin-selection procedure before experimental treatment. Before treatment, spheroid or aggregate cultures with comparable baseline aggregate density and morphology were allocated to the following experimental groups: non-targeting *control* shRNA plus vehicle, non-targeting *control* shRNA plus alisertib, *WSB1*-targeting shRNA plus vehicle, or *EZH2*-targeting shRNA plus alisertib. Alisertib (MLN8237), an Aurora kinase A inhibitor, was prepared as a dimethyl sulfoxide stock solution and freshly diluted into complete spheroid culture medium immediately before use. Based on published prostate cancer and neuroendocrine prostate cancer organoid/cell-line studies [16,17,44], alisertib was used at a final concentration of 100 nM for 7 days, and drug-containing medium was replenished every 3–5 days during treatment. Vehicle-treated cultures received an equivalent final concentration of dimethyl sulfoxide. All comparisons within each model were performed using the same three-dimensional culture plate format, medium composition, and treatment schedule. Enzalutamide-resistant models were maintained under the same continuous enzalutamide condition throughout the experiment. At the experimental endpoint, treatment responses were evaluated by bright-field microscopy, quantitative spheroid or aggregate counting, and immunoblotting. Countable aggregates were defined as compact or semi-compact multicellular structures with clearly discernible boundaries. Single cells, cellular debris, necrotic fragments, and loose non-compact clusters were excluded from aggregate counting. Parallel cultures were collected for immunoblot analysis of WSB1, EZH2, cleaved caspase-3, p53, cleaved poly(ADP-ribose) polymerase 1 (PARP-1), and β-actin. β-actin was used as a loading control. Cleaved caspase-3 and cleaved PARP-1 were evaluated as apoptosis-associated markers, whereas p53 was assessed as a stress-response and tumor-suppressor pathway marker.

### 4.9. Statistical Analysis

Statistical analyses were performed using GraphPad Prism version 6.0 (GraphPad Software, Boston, MA, USA). Data are presented as mean ± standard error of the mean (SEM) unless otherwise indicated. Longitudinal spheroid counts in Figure 1b were analyzed by two-way analysis of variance followed by Dunnett’s multiple-comparisons test, with each time point compared with week 0 within the corresponding culture. Multiple-group comparisons in Figure 2a, Figure 3b,d–f and Figure 6b,d,e were analyzed by one-way analysis of variance followed by Tukey’s multiple-comparisons test. A *p* value < 0.05 was considered statistically significant. Statistical significance is indicated as * *p* < 0.05, ** *p* < 0.01, *** *p* < 0.001, and **** *p* < 0.0001.

## 5. Conclusions

WSB1-dependent PSMA ubiquitination may connect enzalutamide resistance to neuroendocrine-like progression, and therapeutic disruption of the AURKA–WSB1–PSMA axis may suppress refractory prostate cancer spheroid growth while preserving PSMA-directed vulnerability.

## Data Availability

The datasets used and/or analyzed during this study are available from the corresponding author upon reasonable request.

## Abbreviations

3D, three-dimensional; AR, androgen receptor; ATM, ataxia telangiectasia mutated; AURKA, Aurora kinase A; CgA, chromogranin A; CRPC, castration-resistant prostate cancer; DHT, dihydrotestosterone; DMEM, Dulbecco’s Modified Eagle Medium; DMSO, dimethyl sulfoxide; EGF, epidermal growth factor; ENZ, enzalutamide; EZH2, enhancer of zeste homolog 2; FBS, fetal bovine serum; FGF2, fibroblast growth factor 2; FGF10, fibroblast growth factor 10; FOLH1, folate hydrolase 1; HIF, hypoxia-inducible factor; MRI, magnetic resonance imaging; MYCN, MYCN proto-oncogene; NEPC, neuroendocrine prostate cancer; Ni-NTA, nickel-nitrilotriacetic acid; PARP-1, poly(ADP-ribose) polymerase 1; PBS, phosphate-buffered saline; PET, positron emission tomography; PSA, prostate-specific antigen; PSMA, prostate-specific membrane antigen; pVHL, von Hippel-Lindau tumor suppressor protein; shRNA, short hairpin RNA; SOCS, suppressor of cytokine signaling; SYP, synaptophysin; TGF-β, transforming growth factor-β; t-NEPC, treatment-emergent neuroendocrine prostate cancer; Ub, ubiquitin; WT, wild type; WSB1, WD repeat and SOCS box-containing protein 1.
